# The role of β_2_ integrin in dendritic cell migration during infection

**DOI:** 10.1186/s12865-020-00394-5

**Published:** 2021-01-06

**Authors:** Tarfa Altorki, Werner Muller, Andrew Brass, Sheena Cruickshank

**Affiliations:** 1 Faculty of Biology, Medicine and Health, Lydia Becker Institute of Immunology and Inflammation, Manchester Academic Health Science Centre, A.V. Hill Building, The University of Manchester, Oxford Road, Manchester, M13 9PT UK; 2 Present address: Faculty of Medical Applied Sciences, Department of Medical Laboratory Sciences, King Abdul-Aziz University, Jeddah, Saudi Arabia; 3 Faculty of Biology, Medicine and Health, Division of Informatics, Imaging and Data Sciences, Stopford Building, The University of Manchester, Oxford Road, Manchester, M13 9PT UK

**Keywords:** β_2_ integrin, Dendritic cell, Migration, Infection, Immune response

## Abstract

**Background:**

Dendritic cells (DCs) play a key role in shaping T cell responses. To do this, DCs must be able to migrate to the site of the infection and the lymph nodes to prime T cells and initiate the appropriate immune response. Integrins such as β_2_ integrin play a key role in leukocyte adhesion, migration, and cell activation. However, the role of β_2_ integrin in DC migration and function in the context of infection-induced inflammation in the gut is not well understood. This study looked at the role of β_2_ integrin in DC migration and function during infection with the nematode worm *Trichuris muris*. Itgb2^tm1Bay^ mice lacking functional β_2_ integrin and WT littermate controls were infected with *T. muris* and the response to infection and kinetics of the DC response was assessed.

**Results:**

In infection, the lack of functional β_2_ integrin significantly reduced DC migration to the site of infection but not the lymph nodes. The lack of functional β_2_ integrin did not negatively impact T cell activation in response to *T. muris* infection.

**Conclusions:**

This data suggests that β_2_ integrins are important in DC recruitment to the infection site potentially impacting the initiation of innate immunity but is dispensible for DC migration to lymph nodes and T cell priming in the context of *T. muris* infection.

**Supplementary Information:**

The online version contains supplementary material available at 10.1186/s12865-020-00394-5.

## Introduction

Dendritic cells (DC) play a crucial role in orchestrating T cell responses via their ability to prime and activate naïve T cells. DCs are motile cells that must migrate to the site of infection to detect the threat, as well as migrate to the lymph node to prime T cells [1]. Adhesion molecules known as integrins are important for cell migration. Integrins are expressed by a variety of cell types and mediate cellular interaction with the extracellular matrix (ECM) proteins and with other cells through surface-ligand interactions. Accordingly, they facilitate cell movement and stabilize cellular interactions [2]. Integrins play a significant role in mediating a variety of cell signaling processes in homeostasis as well as inflammation [3–5]. One such integrin subunit β_2_ integrin (CD18) is widely expressed by leukocytes such as neutrophils, DCs, macrophages, and T cells [3]. The β_2_ (CD18) integrin subfamily is comprised of four members that share the same β subunit but combine with distinct α subunits: LFA-1 (α_L_β_2_, CD11a/CD18), Mac-1 (α_M_β_2_, CD11b/CD18), (α_X_β_2_, CD11c/CD18), and α_D_β_2_ (CD11d/CD18) [6, 7]. β_2_ integrin plays a crucial role in several immune cell functions such as facilitating intracellular signaling cascades, mediating cytoskeletal rearrangement, recruitment to lymphoid organs and inflamed tissues [8, 9] and adhesion, migration and activation of T cells [7, 10–13]. The role of β_2_ integrin in DC migration is however not fully understood.

The requirement of integrins for DC migration has been debated. Some studies have shown that integrins may not be necessary for DC migration and that DCs lacking functional integrins could migrate through the skin in steady-state conditions [14, 15]. However, the role of integrins could differ depending upon the tissue site or in inflammation, and it remains unclear whether, DCs require integrins to home to tissues such as the gut, during an infectious challenge. In the context of gut infection, early recruitment of DC to the site of infection is driven by epithelial production of chemokines such as CCL5 [16–18] whereas DC migration to the lymph nodes is CCR7- dependant, but the role of β_2_ integrin in this migration is not well characterized [15]. In this study, we explored the role of β_2_ integrin in immune cell recruitment and its function in the context of an infectious immune challenge in the gut. We use the Itgb2^tm1Bay^ model thereafter referred to as Itgb2^mut^ [19]. Itgb2^mut^ mice have a hypomorphic mutation in the cytoplasmic tail domain, which results in impaired β_2_ integrin functions. The mouse phenotype resembles a moderate phenotype form of the leukocyte adhesion deficiency type-I (LAD-I) disease in humans [19].

We hypothesized that the lack of functional β_2_ integrin in the Itgb2^mut^ mice would result in enhanced susceptibility to infection. Studies on the *T. muris* model have shown that a high dose of 200 infective *T. muris* eggs induces a robust Th2 response in resistant mice such as C57BL/6 mice [20, 21], Therefore, to test our hypothesis of enhanced resistance to infection in the absence of functional β_2_ integrin, we infected wild-type (WT C57 BL/6) littermate controls and Itgb2^mut^ mice with a high dose of *T. muris* eggs and determined the response to infection.

## Results

### Itgb2^mut^ mice have a reduction in DC and macrophage recruitment to the infection site in response to *T. muris* infection

**Fig. 1:**
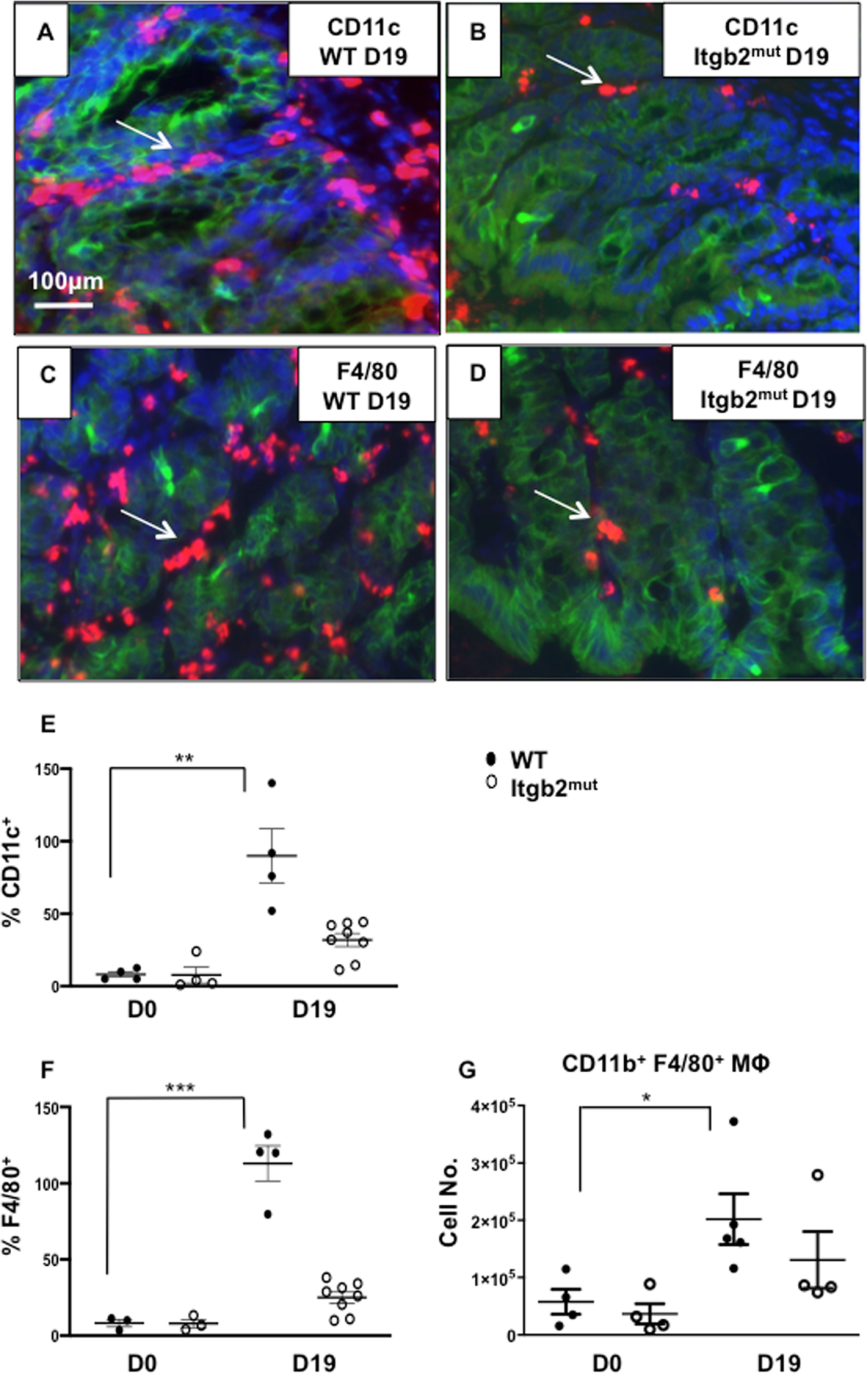
Migration of macrophages in the colon post-infection is affected by the lack of functional β_2_ integrin. Itgb2^mut^ and WT littermate controls were infected with 200 embryonated *T. muris* eggs and colon tissue were analysed by immunohistochemistry staining at day 0 (D0) and day 19 (D19) post-infection(p.i) to identify epithelial cells with cytokeratin (green), CD11c (red) (**a**, **b**) or F4/80 (**c**, **d**) expressing cells (red) and counterstained with DAPI (blue). Shown are representative images at D19 p.i. (**a**, **b**, **c**, **d**) from one of two experiments. The number of (**e**) CD11c^+^ cells and (**f**)  F480^+^ cells were enumerated by calculating the mean of 5 fields/per mouse, *n* = 3–8 mice per time point. CD11b^+^F4/80^+^ macrophages were analysed by flow cytometry, (**g**) Absolute numbers of CD11b^+^F480^+^ macrophages as a proportion of the CD45^+^CD11c^+^MHC^lo^ population were calculated, *n* = 4–5 mice per time point, *(*P* < 0.05), **(*p* < 0.01)

To determine whether the lack of functional β_2_ integrin affected the migration of DCs and macrophages to the colon in response to *T. muris* infection, immunohistochemistry sections from littermate WT controls and Itgb2^mut^ colons were examined in before infection (Day 0) tissue and at Day 19-post infection (p.i) with *T. muris*. This time point of Day 19 (D19) p.i was selected, as it is a time that corresponds to peak time for detection of whether the immune response is polarised to Th2 or Th1 responses. CD11c antibodies staining revealed, that there were a decreased number of CD11c^+^ cells in the Itgb2^mut^ colon p.i, as compared to littermate controls (Fig. 1a, b, e, **p < 0.01). In contrast at Day 0 (D0) there were no differences in the numbers of CD11c cells in the gut between WT and Itgb2^mut^ strains. Since CD11c is not exclusively expressed by DCs and can be expressed by other cells, such as macrophages and eosinophils [22], additional markers were used in combination with CD11c to further characterize the cells as DCs (CD103) or macrophages (F4/80).

The number of F4/80^+^ macrophages was significantly lower in the Itgb2^mut^ colon p.i when compared to WT (Fig. 1c, d, f, ***p < 0.001). This observation was confirmed using flow cytometry, which demonstrated that the absolute numbers of macrophages were increased in the WT colon and not in Itgb2^mut^ colon p.i (Fig. 1g, * *p* < 0.05). This data suggests that a lack of functional β_2_ integrin affects the recruitment of macrophages to the site of infection.

**Fig. 2:**
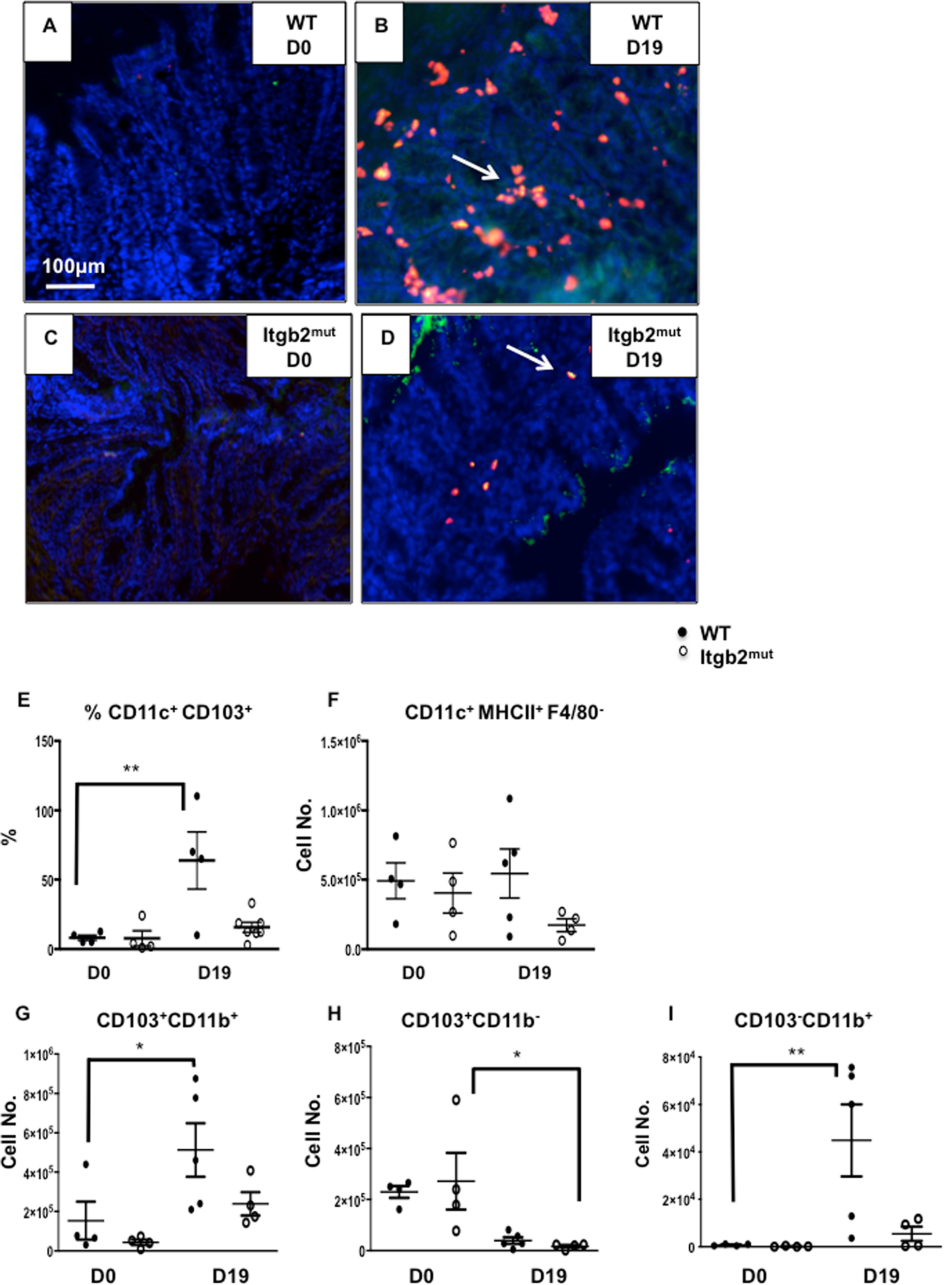
Recruitment of dendritic cells in response to infection was impaired by the lack of functional β_2_ integrin. Itgb2^mut^ and WT controls were infected with 200 embryonated *T. muris* eggs and CD11c^+^CD103^+^ DCs in the colon were assessed by immunohistochemistry using CD11c (red), CD103 (green) and counterstained with DAPI. Representative images of immunohistochemistry staining at day 0 (**a**, **c**), and D19 (**b**, **d**) post-infection in WT and Itgb2^mut^ mice from one of two experiments. (**e**) CD11c^+^CD103^+^ DCs in the colon were enumerated and the mean of 5 fields of view per mouse was plotted, n = 4–8 mice /time point. (**f**, **g**, **h**, **i**) Colonic DCs were isolated both from WT and Itgb2^mut^ mice at D0 and D19 post infection, data were analysed by flow cytometry to assess DC subsets in the colon. Absolute numbers of CD45^+^ cells were calculated for (**f**) CD11c^+^MHCII^+^F4/80^−^, (**g**) CD103^+^CD11b^+^, (**h**) CD103^+^CD11b^−^, and (**i**) CD103^−^CD11b^+^ (I) DC subsets in the colon, *n* = 4–5 mice/time point, *(*P* < 0.05), **(*p* < 0.01)

Immunohistochemistry also showed that the numbers of CD11c^+^CD103^+^ DCs were similar between WT and Itgb2^mut^ mice before infection (Fig. 2a, c, e). In response to infection, there was a marked increase in the number of CD11c^+^CD103^+^ DCs in infected WT colon that was not seen in the infected Itgb2^mut^ colon in which DC numbers remained low (Fig. 2b, d, e, **p < 0.01). These data suggest that the recruitment of this particular subset of DCs was affected by the lack of functional β_2_ integrins during infection but not in steady-state. To further characterize DCs in the gut and draining lymph nodes, DC subsets were analyzed using flow cytometry.

In addition to the variation in the number of cells between WT and Itgb2^mut^ colons at D19 p.i, we have observed a different distribution of CD11c^+^ and CDF4/80^+^ cells (Fig. 1a-d) and CD11c^+^CD103^+^ cells (Fig. 2b, d) between both strains. Immunostaining showed DCs and macrophages were located both in the lamina propria and adjacent to the colonic epithelium in the WT colon at D19 p.i. However this was not mirrored in the Itgb2^mut^ colon as the cells were mostly found in the lamina propria.

Lamina propria cells were further analyzed using flow cytometry pre- and post-infection with a gating strategy to identify the different gut DC subsets shown in (supplementary Figure 1A). In response to infection, the number of CD11c^+^MHCII^+^ F4/80^−^DCs in the Itgb2^mut^ colon remained unchanged pre- and post-infection (Fig. 2f), numbers of colonic CD11c^+^ MHCII^+^ F4/80^−^ DCs were more variable in the WT mice p.i, but trended to be higher. An analysis of the DC subsets by flow cytometry revealed, akin to the immunohistochemistry data, that the majority of the DCs in the colon were CD103^+^CD11b^+^ DCs (Fig. 2g). CD103^+^CD11b^+^ DCs increased twofold to threefold in the infected WT colons when compared to Day 0 WT (Fig. 2g, *p < 0.05). In contrast, this subset did not increase in the Itgb2^mut^ colon p.i when compared to D0 and infected WT, and although reduced numbers were detected in the mutant colon p.i compared to infected WT colon this reduction was not significant (Fig. 2g).

The number of CD103^+^CD11b^−^ DCs decreased in response to the infection relative to D0 mice in both WT and Itgb2^mut^ colons at similar levels (Fig. 2h, *p < 0.05). The absolute number of the CD103^−^CD11b^+^ subset of DCs increased moderately in the WT colons compared to the D0 and infected WT (Fig. 2i, p* < 0.05). Taken together this data suggests that the main DC subset found in the gut in this infection model was the CD103^+^ CD11b^+^ DC subset and their frequency was reduced in the absence of functional β_2_ integrin. To check whether there was a change in DC maturation, expression of the co-stimulatory molecule CD86 on DCs was compared using the mean fluorescence intensity (MFI) pre and post- infection. Results show that CD103^+^ CD11b^+^ DCs had higher expression of CD86 at Day 5 (D5) p.i compared with naïve (D0) mice in the WT colon suggesting they were activated and becoming mature (supplementary Fig. 2A). In contrast, the same subset in the Itgb2^mut^ mice at Day 5PI had relatively low CD86 suggesting they were still immature (supplementary Fig. 2 A). By D19 p.i CD86 levels on gut DCs were similar to naïve (D0) levels suggesting the DCs present in the gut at this time point were immature. Expression of CD86 on the CD103^+^CD11b^−^ DCs at D5 p.i was variable and there were no significant differences seen in the WT or Itgb2^mut^ mice compared to cells from uninfected (D0) mice (supplementary Fig. 2B). CD103^−^ CD11b^+^DCs did not show any up regulation of CD86 in either WT and Itgb2^mut^ colonic cells (supplementary Fig. 2C).

Given the effect of β_2_ on immune cell recruitment to the site of infection we next investigated whether this might impact on recruitment to the lymph node.

### The lack of functional β_2_ integrin did not impair CD103^+^ DC migration to the mesenteric lymph node (MLN) in response to infection

**Fig. 3:**
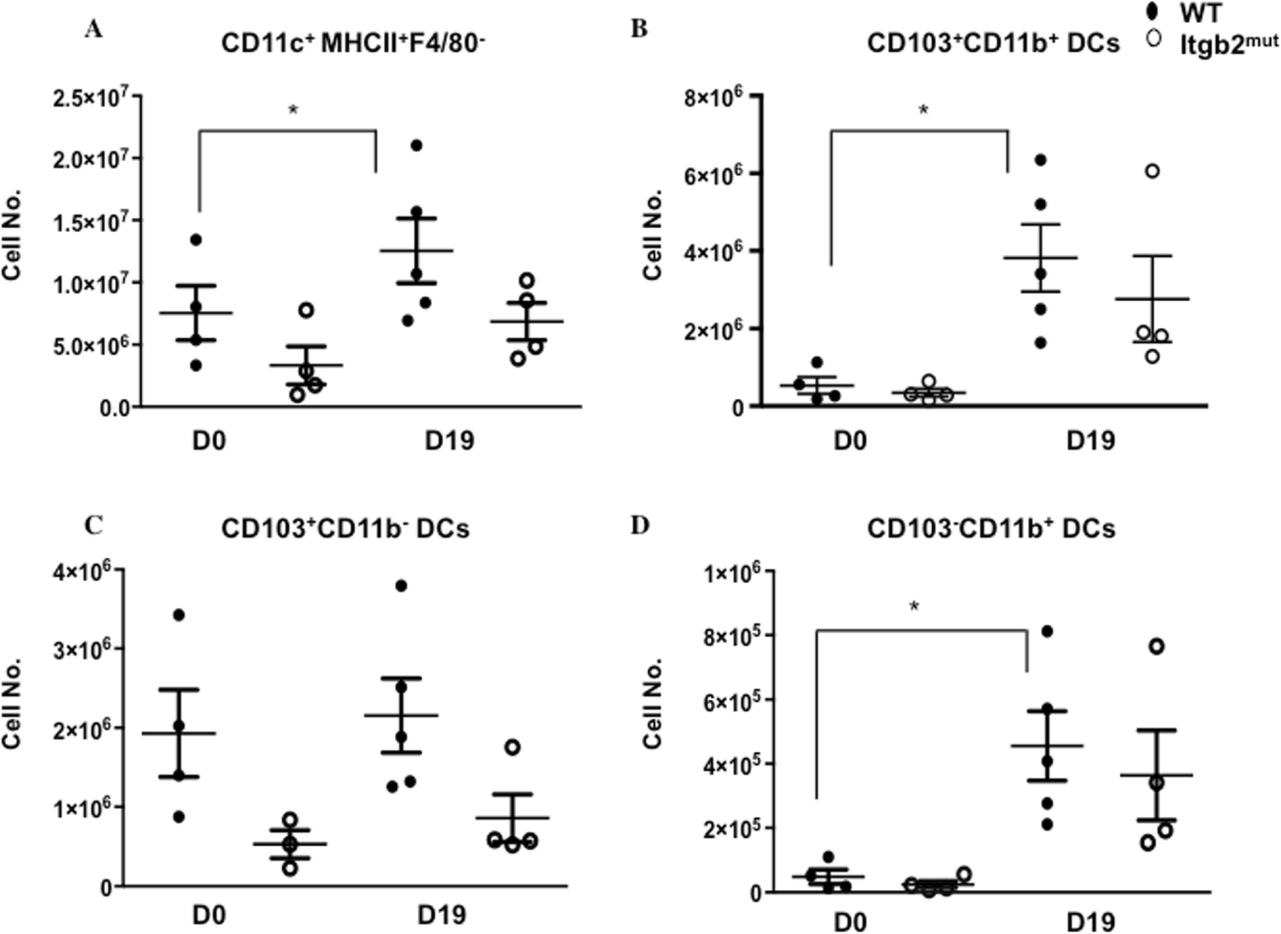
Dendritic cell migration to the MLN in response to infection was unaffected by the lack of functional β_2_ integrin. DCs were isolated from the MLN of Itgb2^mut^ mice, and littermate control (wild type) at D0 and D19 post-infection were analysed by flow cytometry to look at DC subsets in the MLN. Absolute numbers of CD45^+^ cells were calculated for (**a**) CD11c^+^MHCII^+^F4/80^−^, (**b**) CD103^+^CD11b^+^, (**c**) CD103^+^CD11b^−^, and (**d**) CD103^−^CD11b^+^ DC subsets in the MLN at day 0 and post-infection, *n* = 4–5 mice/time point, *(*P* < 0.05).

A gating scheme to identify the DC subsets is shown in (supplementary Figure 1A). DC subsets were assessed in the MLN from naïve (D0) mice and p.i. The absolute numbers of CD11c^+^MHCII^+^ F4/80^−^ DCs in the MLN showed no significant changes in the overall DC numbers in Itgb2^mut^ mice at either time point, however, this was slightly increased in WT p.i compared to day 0 (Fig. 3a, *p < 0.05). The majority of the DCs detected in the MLN at D19 p.i were CD103^+^ migratory DCs in both WT and Itgb2^mut^ (Fig. 3b, c). The CD103^+^ DCs were further analyzed to identify the DC subset, and the absolute numbers showed an overall threefold increase in CD103^+^CD11b^+^ DCs in WT MLNs p.i as compared to day 0 (Fig. 3b, *p < 0.05). There was no significant difference in the numbers of CD103^+^CD11b^+^ or CD103^+^CD11b^−^ DCs in WT and Itgb2^mut^ MLN between strains p.i., yet there was a trend for a reduction of these two subsets in the Itgb2^mut^ MLN (Fig. 3b, c). A small proportion of the CD103^−^ CD11b ^+^ DCs were also present in the MLNs and an increase in this subset was seen p.i in both WT and Itgb2^mut^ as compared to D0 (Fig. 3d, *p < 0.05). Collectively, these data suggest that DC migration to the MLN was not impaired in the Itgb2^mut^ mice in response to the infection, suggesting that T cell priming would be unaffected in the Itgb2^mut^ mice.

### Adaptive immune responses to *T. muris* were unaltered in Itgb2^mut^ mice post-infection

**Fig. 4:**
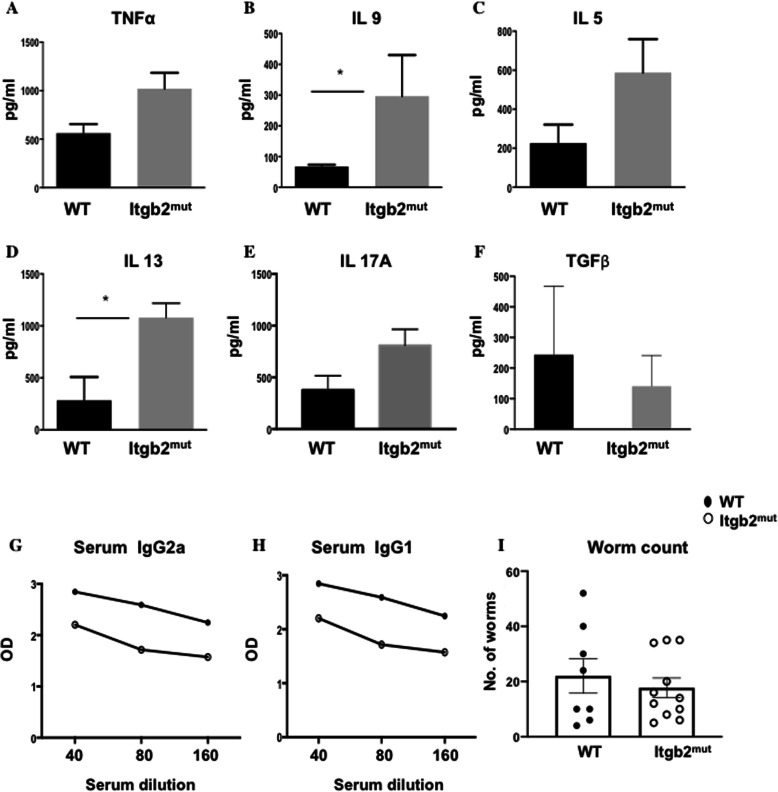
The effector immune response to *T. muris* was unaffected in the absence of functional β_2_ integrin. MLN cells were analysed from *T. muris* infected mice at D19 post-infection and re-stimulated in vitro with 50 μg E/S antigen for 48 h. Supernatants were analysed by CBA for (**a**) TNF-α, (**b**) IL-9, (**c**) IL-5, (**d**) IL-13, (**e**) IL-17A, (**f**) and TGF- β. Graphs show the mean of n = 4–8 mice. (**g**) *T. muris* specific serum IgG2a and (**h**) IgG1 were analysed using ELISA. **i** Worm burden was enumerated at D19 post-infection, *n* = 4–8 mice/time point, *(*P* < 0.05)

**Fig. 5:**
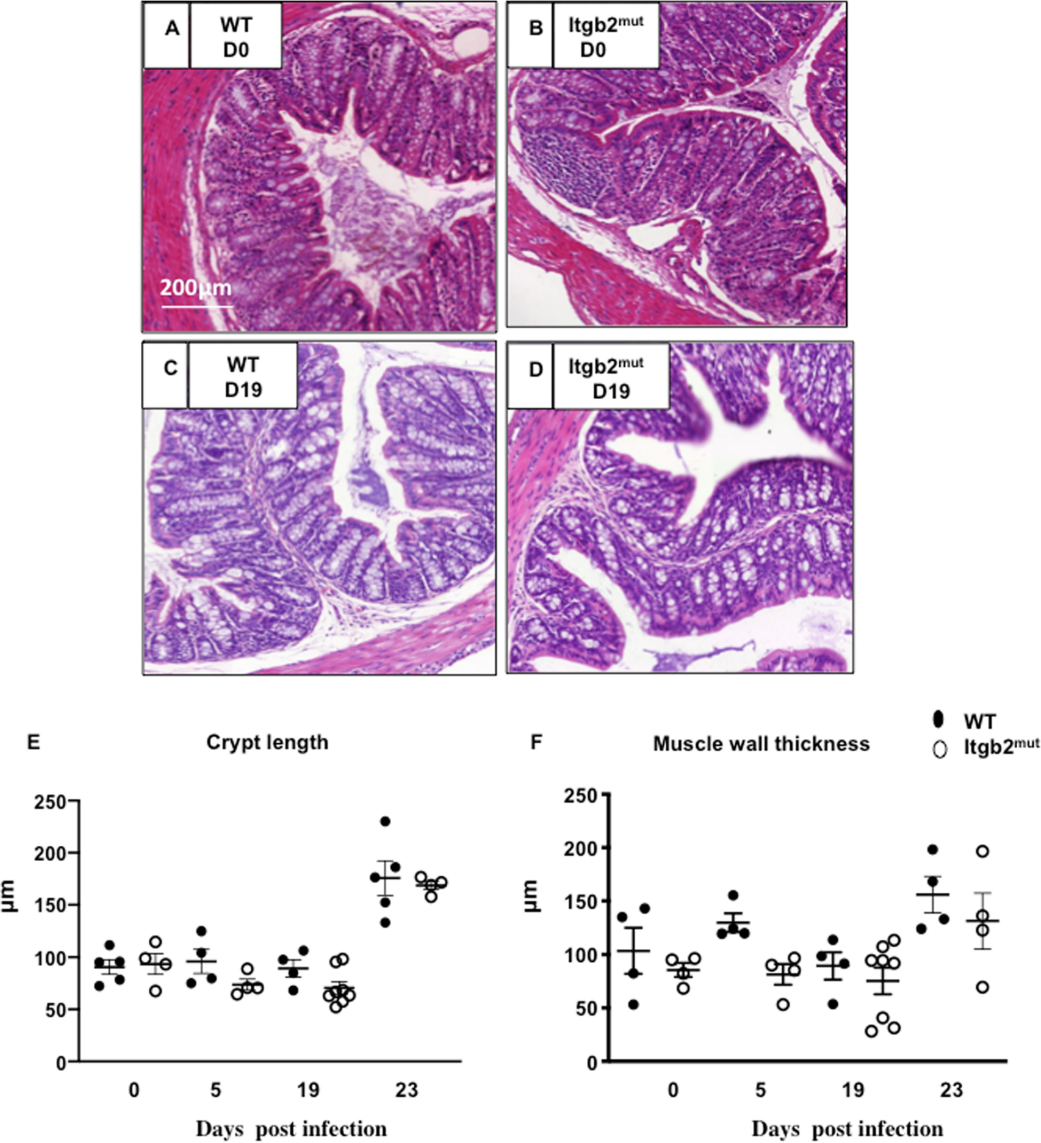
Colon pathology was unaltered in WT and Itgb2^mut^ mice pre- and post- infection with *T. muris.* Itgb2^mut^ and WT littermate controls mice were infected with 200 embryonated *T. muris* eggs and harvested at Day (D0) (**a**, **b**), D5, and D19 (**c**, **d**) post-infection and colon pathology was assessed by H&E stain. Representative images of histology from D0 and D19 are shown from one of two experiments**.** (**a**-**d**). (**e**) Average crypt length and (**f**) muscle wall thickness was quantified as the mean of 20 measurements/tissue using Image J software, *n* = 4–8 mice/per time point.

To test whether the changes in DC dynamics to the infection site impacted the development of the adaptive immune response, we assessed specific T cell responses in the MLN. We also investigated IgG, worm burden, and pathology. MLN cells were stimulated in vitro with parasite excretory/secretory (E/S) antigen for 48 h and the cytokine response analyzed. There were no significant differences between the WT and Itgb2^mut^ MLN cell responses in the levels of IL-6, IL-10, IFN-γ, IL-12p70, and TGF- β. However, there was a trend for elevated levels of IL-13, IL-9, IL-5, IL-17A, and TNF-α with IL-9 and IL-13 significantly increased in the Itgb2^mut^ MLN when compared to WT (Fig. 4a-f *P < 0.05), which is indicative of a robust Th2 response. Consistent with such a Th2 dominated immune response; we observed a higher IgG1 response as compared with IgG2a, with both strains of mice generating *T. muris-*specific IgG1 (Fig. 4g, h). The overall levels of IgG were generally lower in the Itgb2^mut^ mice, but this was not significant (Fig. 4g, h). Collectively this data suggested that the Th2 response was, as anticipated, intact in the Itgb2^mut^ mice, therefore we next assessed worm burden. In line with a robust Th2 response, there was no significant difference in the worm burden between the strains (Fig. 4i). Finally, we investigated whether the reduced magnitude of DC recruitment in the colon in response to infection was associated with altered colon pathology. Data showed no significant changes between the colons of WT and Itgb2^mut^ mice in crypt length measurement or muscle wall thickness during infection (Fig. 5). However, at D23 p.i, there was an increase in crypt length and muscle wall thickness in both strains (Fig. 5e, f) suggesting the lack of DC recruitment had not impacted pathology.

**Fig. 6:**
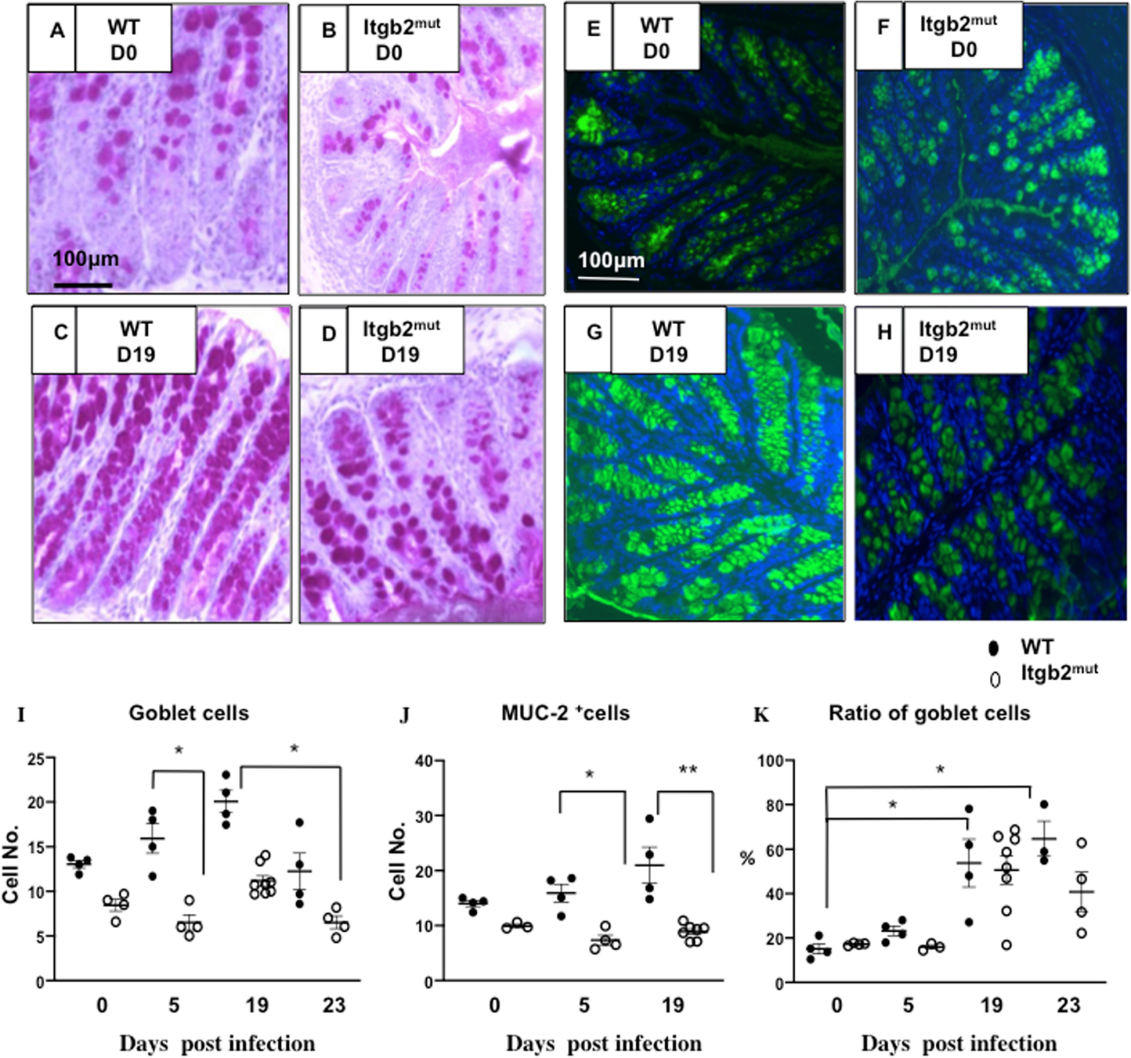
Itgb2^mut^ mice did not develop infection induced goblet cell hyperplasia in response to infection with *T. muris* and had fewer MUC-2 expressing cells in the colon than littermate controls in response to infection. Itgb2^mut^ and WT littermate controls mice were infected with 200 embryonated *T. muris* eggs and harvested at Day 0 (D0) (**a**, **b**), D5, and D19 (**c**, **d**) post-infection. Representative images of colons taken from one of two experiments that were assessed by PAS staining for the quantification of goblet cells (**a**-**d**) or Muc2 to identify mucin-2 expressing cells (**e**-**h**). Images were analysed using Image J software and (**i**) the number of goblet cells, (**j**) the number of Muc-2 expressing cells, and (**k**) the ratio of goblet to epithelial cells were calculated as the mean of 20 measurements /tissue section, *n* = 4–8/time point. *(*P* < 0.05), (**p < 0.01)

Goblet cells are known to have a protective role in *T. muris* infection via the secretion of mucins [23]. To assess the goblet cells, we performed periodic acid Schiff (PAS) staining and enumerated the goblet cells in the colon pre- and post-infection (Fig. 6a-d). Although no significant differences were found in the number of goblet cells of uninfected WT and Itgb2^mut^ mice, there was a trend of reduced goblet cells in the Itgb2^mut^ mice (Fig. 6a, b, i). WT mice developed a moderate goblet cell hyperplasia p.i (Fig. 6c, i, *P < 0.05), however, there was no such increase in the Itgb2^mut^ mice (Fig. 6d, i, **P < 0.01). Muc2 is the major mucin in the colon [24, 25] therefore; we also assessed the expression of Muc2 in Itgb2^mut^ and WT mice (Fig. 6e-h). We observed fewer Muc2 positive cells in the colon of the Itgb2^mut^ mice p.i. compared with WT (Fig. 6g, h, j **p < 0.01) suggesting that Itgb2^mut^ mice did not have an infection-induced increase in the goblet cells and Muc2 secretion. However, evaluation of the ratio of goblet to epithelial cells showed there was a similar goblet to epithelial cells ratio between WT and Itgb2^mut^ colons during *T. muris* infection (Fig. 6k).

Collectively our data show that in the context of a Th2 response in the colon, β_2_ integrin is involved in DC recruitment to the site of infection but not trafficking of DCs to the lymph nodes and establishment of Th2 immunity and subsequent resistance to infection.

## Discussion

Over the last 10 years, research using models such as transgenic knockout models have provided major progress in understanding the role of integrins. However, questions remain regarding the role of integrins in leukocyte migration and whether there is an absolute requirement for them for effective DC migration during infection. DCs were shown to migrate interstitially within the skin and lymph node without the need for integrins under steady-state conditions [14]. Furthermore, β_2_ integrin was found to restrict DC migration to lymph nodes during homeostasis [8]. In agreement with these observations, we observed no changes in the frequency of DCs in the MLN in both wild type and Itgb2^mut^ mice before infection suggesting no role in steady-state trafficking to lymph nodes. Furthermore, although we saw trend for a reduction, we found no significant changes between the proportion of DC subsets in the MLN in WT and Itgb2^mut^ mice post- infection suggesting that DC migration to the MLN in infection was also not dependent on functional β_2_ integrin. CCR7 plays a critical role in DC migration to the MLN in the gut and skin during steady-state and inflammation [15, 26], thus chemokine signaling may be more important for this directional movement of DCs to the lymph nodes. Future studies could address the relative contributions of β2 integrin and chemokine signaling in recruitment of DCs to lymph nodes.

This paper has focused on studying the role of β_2_ integrin in DC migration to the site of infection and subsequently the efficiency of an immune response to infection. Observing DC and macrophage migration to the colon, the scenario was different from that seen in the MLN. Flow cytometry and immunostaining data showed a  significant decrease in DCs and macrophages in the gut in response to infection in the absence of functional β_2_ integrin. This data suggests DC and macrophage migration to the site of infection is impaired in the absence of functional β_2_ integrin. One possibility for this observation might be that cells are not able to enter the tissue and instead accumulate in lymphoid organs such as the spleen. Splenomegaly was observed in all Itgb2^mut^ mice post-infection but MLNs were comparable with WT. Another possibility is that the reduction in cell number was due to a reduction of DC and macrophage precursors to the tissue site. Pre-DCs are important for the generation of cDCs during steady-state conditions [27–29]. In contrast, in inflammation, inflammatory DCs are thought to be largely derived from monocytes [30]. Monocytes also play a critical role in the development of inflammatory macrophages [29–31]. Low β_2_ expression is associated with monocytopenia in the peripheral blood and bone marrow [32]. This monocytopenia had impacts on the accumulation of monocyte-derived macrophages and monocyte-derived DCs in the lungs early after infection, suggesting that β_2_ plays a role in regulating monocyte hematopoiesis and is needed for transendothelial migration of monocytes [32]. The observation that both macrophages and dendritic cells numbers were reduced in inflammation but not in steady-state does suggest a role for β_2_ integrin in promoting monocyte recruitment to the colon and/or extravasation of monocyte precursors to during infection that in turn impacts on the development of inflammatory dendritic cells and macrophages. β_2_ integrin has also been reported to mediate macrophage retention at inflammatory sites [11], therefore it may be that monocyte recruitment is unaffected but the macrophages and DCs are not retained at the site of infection which will be interesting to address in future studies. Similar to our observations of unaltered DC migration to lymph nodes, other infection studies have also observed that DC migration to the lymph nodes was not affected by the lack of β_2_ integrin [33, 34], and it is believed that this process is CCR7 dependent [15]. There are both residents and recruited populations of dendritic cells in the lymph nodes and through our flow cytometry panel suggested no clear differences in overall DC numbers between WT and mutant mice in the nodes, the possibility is that the balance of recruited versus residents cells may be altered. However, T cell responses were not diminished implying that sufficient DC had been trafficked to provoke immune responses. It may be that more acute models of inflammation coupled with intra-vital imaging or the use of approaches such as mesenteric lymphadenectomy and thoracic duct cannulation to identify intestinal migratory DC subsets could better resolve the question of recruitment of DCs to the nodes [35]. It will be also interesting to further assess the contribution of β_2_ in monocyte recruitment and cell retention in the gut and the development of cell-specific knockouts would be invaluable for this purpose for both in vivo and in vitro studies. Such tools could also enable further functional assessment on integrin functionality in colonic dendritic cells.

In the context of gut infection, early DC recruitment to the site of infection has been associated with resistance to infection, which is thought to be due to DCs receiving the appropriate maturation signals in a timely fashion that enables them to prime the appropriate T cell response. Delay of DC maturation will affect DC priming T cells in the MLN, which may result in a delay in the development of the T cell response [16–18]. Therefore, the reduction of DCs in the inflamed gut might be anticipated to impact on subsequent immunity. However, there was no difference in susceptibility to the infection to *T. muris* in WT and Itgb2^mut^ mice and there was no clear reduction in numbers between DCs in the nodes between strains. Whereas DCs appear to be critical for Th1 development [36], several papers have suggested that DCs are not essential for Th2 immunity and that other cells such as basophils or ILC2s are important for resistance to Th2 infections [37–39]. Indeed, basophils have been shown to play a protective role by inducing strong Th2 immunity during *T. muris* infection [40, 41]. Thus, the impact of this reduced DC recruitment on the gut in this infection may not markedly impact immunity in the context of a Th2 immune response however it would be interesting to assess in future work if this is true in the context of small intestinal infection or Th1 responses.

β_2_ integrin is thought to play a role in T cell function. Integrins on DCs and other APCs regulate the T cell response [42–44]. β_2_ integrin was shown to be essential for T cell migration to the MLN but dispensable for T cell priming by DCs [45, 46]. WT and Itgb2^mut^ mice expelled *T. muris* worms equally and Th2 associated cytokine responses were detected in WT and Itgb2^mut^ MLN. This data would appear to confirm that β_2_ integrin on DCs was not needed for T cell priming. As DCs lacking functional β_2_ integrin are fully capable of priming T-cells, and β_2_ expression on DCs can be inhibitory for T cell activation [46, 47]. We propose that β_2_ integrin might have a role in modulating the development of Th2 responses. We did see slightly higher levels of Th2 associated cytokines in our infection model in the absence of β_2_ integrin. One possible mechanism may be that responses are less attenuated in the absence of β_2_ integrin as the integrin has been associated with the induction and modulation of Treg responses [13, 48]. In future studies therefore it may be interesting to assess the role of β_2_ integrin in the development of Treg populations in the context of gut infection and inflammation. Additionally, although it was not significant, IL-17A production increased in the Itgb2^mut^ MLN compared to the WT. IL-17 is increased in CD11b deficient mice, which induced Th17 cell differentiation and prevented the mice developing oral tolerance [49]. The role of β_2_ in regulating IL-17 has been suggested in other studies [43, 50]. TGF- β is also involved in Th17 differentiation  and levels were similar in  WT and Itgb2^mut^ at D19 p.i. and thus the impact on Th17 cells in this model might be limited. It would also be interesting to assess Th1 and Th2 polarisation more fully in other models of infection and inflammation as C57BL/6 mice are resistant to *T. muris* infection and the changes in T cell cytokines and antibody had no impact on resistance to infection or gut pathology. β_2_ integrin was found to be essential in regulating the immune response in the skin and lymph node during skin inflammation and dysfunctional β_2_ was associated with increased Th1 responses [43]. The use of cell-specific knockout strains to assess both DC function in T cell priming and T cell function would be invaluable in further dissecting mechanisms for future study.

A key difference between the β_2_ integrin deficient mice and WT was in hyperplasia of goblet cells. PAS and Muc2 staining of Itgb2^mut^ mice revealed that there was no infection-induced hyperplasia in the mutant mice and no increase in Muc2 levels at D19 p.i. compared with WT. Goblet cell hyperplasia is associated with immune resistance against nematode infection [24, 25, 51]. Th2 cells induce IL-4, IL-9, and IL-13, which promote epithelial cell mucus production, which protects the host’s epithelial barrier and promotes worm expulsion [52, 53]. Although IL-9 and IL-13 production was increased in Itgb2^mut^ MLN, mucus production did not appear to be enhanced compared with WT however worms were still expelled. Furthermore, the ratio of goblet cells to epithelial cells was similar between strains. Thus, it may be the levels of Th2 associated cytokines were insufficient to alter mucus production but it raises the interesting possibility of a link between β_2_ integrin and the mucus layer. Data does suggest the intriguing possibility that β_2_ plays a role in the function and proliferation of goblet epithelial cells, integrins such as β_1_ have been found to control the fate and function of cells by controlling their proliferation and differentiation [54]. Intestinal goblet cells can transport luminal antigens to CD103^+^ DCs [55] and therefore a lack of goblet cell hyperplasia, along with dysfunctional DCs may impact DC antigen acquisition that would impede the immune response. However, if this were the case, worm burden data and pathology results would be impacted and this was not the case.

In summary, our findings highlighted the role of β_2_ integrins in migration to the tissue sites during a Th2 driven infection adding to the body of literature on the role of integrins in cell migration and function.

## Methods and materials

### Mice

C57BL/6 Itgb2^tm1Bay^ mutant mice referred to (Itgb2^mut^) carry a mutation in the cytoplasmic tail of CD18 and were described previously [19]. Mice were bred as heterozygous pairs and were fed irradiated food and water. All mice were kept in well-ventilated cages, and had free access to water and standard chow and were used at 8–12 weeks of age. Mice were maintained on a 12 h light-dark cycle at 21–23 °C in the Biological Services Unit (BSU), University of Manchester. All animal experiments and procedures were approved by the University of Manchester Animal Welfare and Ethical Review Board and performed by the Home Office guidelines scientific procedures Act 1986 (revised 2013).

### *Trichuris muris* infection

Itgb2^mut^ and wild type littermate controls were infected with approximately 200 embryonated *T. muris* eggs by oral gavage. *T. muris* eggs were prepared and maintained as described [56]. Mice were sacrificed at days 0, 5, and 19 post-infection We selected three time points as the focus for study based on previous work in the model- Day 0, D5, and D19 [17, 57] as these are timepoints that coincide with peak dendritic cell recruitment to the colon [17] (D5) and colonic inflammation and T cell responses (D19) [41, 57, 58]. Mice were selected for experimental use per ARRIVE guidelines. Briefly, mice to be infected were randomised from 3 to 8 litters per experiment using a spreadsheet, the sample size was selected based upon previous work and all experiments were blinded.

All experiments were performed on littermates to control for variation in the microbiome and environment and each experiment we randomised mice from 3 to 8 litters. However, the use of littermate controls meant there was some variance in group sizes between experiments with at least 4 mice/ experiment. All experiments except flow cytometry were performed on at least two sets of experiments.

### *T. muris* worm burden count

The numbers of *T. muris* worms were counted in the caecum post-infection (p.i). The worms were counted in the fecal matter as well as caecum tissue as described previously [56].

### Isolation of cells from the colon

Colonic cells were harvested at autopsy and digested in RPMI media containing 5% L-glutamine, 5% streptomycin, 10% fetal bovine serum (Sigma Aldrich, Dorset, UK), dispase (0.5 mg/ml), and collagenase type VIII (100 U/ml) from *Clostridium histolyticum* (Sigma-Aldrich). Cells were ten incubated in a rotator under gentle agitation at 37 °C for 2 h. Digested tissue was forced through a 70 μm cell strainer (ThermoFisher Scientific, Cheshire, UK), the collected suspension was then centrifuged at 900 g for 5 min and re-suspended in 10 ml of 40% percoll overlaid onto 5 ml 80% percoll solution (GE Healthcare, Buckinghamshire, UK) and centrifuged at 1000 g for 20 min. The cells at the gradient interface were carefully collected, washed in RPMI, centrifuged at 500rcf for 5 min, and re-suspended at a density of 2 × 10^6^ cells/100 μl in FACS buffer (PBS containing 5% bovine serum albumin (BSA) and 0.02% Sodium Azide (NaN3), all Sigma Aldrich).

### Stimulation of MLN cells

MLN from mice infected with *T. muris* was taken at post mortem and the MLN cells prepared as described previously [17], and 5 × 10^6^ MLN cells/ml were plated in a 96 well culture plate and stimulated with *T. muris* parasite excretory-secretory antigen (E/S, 50 μg/ml, provided by Professor Else, University of Manchester) and incubated at 37 °C, 5% CO_2_ for 48 h. At 48 h the cells were centrifuged at 250 g for 5 min, the supernatants collected and stored at − 20 °C until analysed by cytometric bead array (CBA).

### Flow Cytometry

Cells at (1 × 10^6^/ml) were collected and re-suspended in 300 μl FACS buffer. The cell suspension was then stained for flow cytometry analysis. Fc receptors were blocked using an anti-CD16/32 antibody (2 μg/ml), (eBioscience, Cheshire, UK). The cells were then washed and stained with FITC-anti-CD45 (1 μg/ml), AlexaFluor700-anti CD11c (1 μg/ml), APC-anti-F4/80(1 μl/ml), PE-antiCD86, (2 μg/ml), (all eBioscience), and pacific blue-anti-MHCII (1 μg/ml, BioLegend, London, UK), APC-cy7-anti-CD11b (1 μg/ml, BD, Plymouth, UK), PercP/Cy5.5-anti-CD103 (2 μg/ml) antibodies, and V510 (viability stain, 1 μg/ml).

Single stain controls were prepared using beads or spleen cells, negative controls were prepared using unstained cells. Cells were acquired by flow cytometry on the BD LSRII (BD) and the data were analyzed and visualized using FlowJo™ flow cytometry software (Ashland, OR: Becton, Dickinson and Company; 2019, Berkshire, UK; https://www.flowjo.com/).

### Cytometric bead Array (CBA)

The levels of IL-6, IL-5, IL-2, IL-10, IL-13, IL-12p70, IFN-γ and, TNF-α in ES stimulated MLN cells were determined using the Beckton Dickinson Cytometric Bead Array (CBA) Mouse/Rat Soluble Protein Flex Set system (BD) according to the manufacturer’s guidelines. Samples were analysed using a MACSQuant analyzer (Miltenyi Biotec Ltd., Bisley, UK) and FCAP Array software (BD, Berkshire, UK).

### Enzyme-linked immunosorbent assay (ELISA)

*T. muris* specific IgG1 and IgG2a antibody responses were investigated in the serum of day 19 infected mice. ELISA plates were coated with 5 μg/ml *T. muris* ES antigen diluted in carbonate bicarbonate buffer (15 mM Na_2_CO_3_, 35 mM NaHCO_3_ adjusted to pH 9.6) and incubated overnight at 4 °C. Plates were washed with PBS Tween (PBS containing 0.05% Tween20, Sigma-Aldrich) and blocked with 3% BSA in PBS Tween. Serum was diluted 1:20–1:2560 in PBS Tween, added to the plate at 50 μl/well, and incubated for 1 h at 37 °C. Plates were washed and incubated with 50 μl/well of biotinylated rat anti-mouse IgG1 (BD Biosciences, Oxford, UK) or IgG2a (BD Biosciences) for 1 h at room temperature. After washing, plates were incubated with 75 μl/well streptavidin β peroxidase (Roche Diagnostics GmbH, Germany) for 1 h at room temperature. Plates were then washed and developed with 100 μl/well of 3,3′, 5,5′ tetramethylbenzidine (Ultra TMB ELISA substrate, Thermo Fisher Scientific). The color development was stopped by adding 100 μl/well of 2 N sulphuric acid (R&Dsystems, Abingdon, UK). Absorbance was read using a Dynex MRX11 plate reader (Dynex Technologies, West Sussex, UK) at 405 nm with a reference of 490 nm.

### Histology

Distal colon snips (~ 3 mm in length) were fixed in NBF (4% neutral buffered formalin, Sigma-Aldrich) for 24 h or in Carnoy’s solution in methanol-Carnoy’s solution to preserve mucus integrity [59]. Samples were left in Carnoys for 1–7 days. The fixed samples were placed in histology cassettes and transferred into 70% ethanol for processing using a Microm STP 120 Tissue Processor (Microm International, Walldorf, Germany). Briefly, tissues were placed in 70% ethanol for 15 min, 90% ethanol for 30 min, 95% ethanol for 30 min, two changes of 100% ethanol for 60 min, 100% ethanol for 20 min, xylene for 15 min, two changes of xylene for 30 min (all at 40 °C), and fribowax pastillated wax (BD) for 1 h at 60 °C. The processed samples were then mounted in wax blocks using a Micro EC-350 embedding system (Microm International) and 5 μm serial-sections were cut using a Microm HM325 microtome (Microm International). The sections were floated on warm water and transferred to glass slides and dried for staining.

### Hematoxylin and eosin staining

The formalin-fixed tissue sections were dewaxed in Citroclear (HD supplies, Aylesbury, UK) for 15 min and rehydrated through absolute alcohol to 70% alcohol (1 min in each concentration of alcohol). The sections were briefly washed in distilled water and stained in Harris Haematoxylin solution (Sigma-Aldrich) for 4 min. After staining, the sections were washed in running tap water for 1 min followed by differentiation in 1% acid alcohol for 10 s. The sections went through washing and bluing in running tap water for 5 min before eosin staining. After staining using eosin solution (Sigma-Aldrich) for 30 s, the sections were washed in tap water for 1 min. Dehydration was performed whereby the sections were taken through changes of 70% alcohol through to absolute alcohol. Before mounting the sections were immersed in Citroclear (HD supplies) for 30 s. The sections were finally mounted with DEPEX mounting media (Thermo Fischer Scientific, Hemel, UK) and sealed. Slides were viewed using a Nikon Eclipse E400 microscope (Nikon, Japan) equipped with lenses at × 20 and × 40 magnification. The average of villus heights was determined by measuring the length of 10 to 15 villi from three different fields of view per specimen using NIH Image software (ImageJ, version 1.45 s, https://imagej.nih.gov/ij/docs/index.html). All slides were analysed in a blinded fashion.

### Goblet cell staining

The mucins in goblet cells were stained with 1% alcian blue (Sigma-Aldrich) in 3% acetic acid (Sigma-Aldrich, pH 2.5) for 5 min, washed in distilled water, and treated with 1% periodic acid for 5 min (Sigma-Aldrich). Sections were washed in distilled water, then tap water for 5 min and rinsed in distilled water. Sections were then treated with Schiff’s reagent (Sigma-Aldrich) for 15mins. Slides were washed again as before being counterstained with Mayer’s hematoxylin (Sigma-Aldrich). Slides were washed in tap water, dehydrated (as described in section 2.9), mounted and coverslipped using DEPEX mounting media (BDH Laboratory Supplies). The slides were scanned by the panoramic viewer system and the goblet cells were enumerated in 20 randomly selected crypts/section and scored blinded.

### Immunofluorescence

Colon fragments from the proximal colon were snap-frozen in optimal cutting compound (OCT, Raymond A Lamb, Eastbourne, UK) and stored at − 80 °C for at least 24 h. The OCT embedded tissue was cut into sections at 4–6 μm thickness using a Leica CM-1100 cryostat (Leica Biosystems, Linford Wood, UK) placed onto charged slides (Thermo Scientific) and stored at − 80 °C until assayed.

Cryo-preserved tissue sections on slides were fixed using 4% paraformaldehyde solution for 5 min followed by washing in washing buffer (PBS with 0.05% BSA) for 5 min. The tissue sections were blocked using tryamide blocking reagent (Perkin Elmer, Cambridge, UK) at room temperature for 30 min, washed three times in washing buffer, blocked again using avidin/biotin blocking kit (Invitrogen) at room temperature for 15 min each, washed three times in washing buffer, followed by incubation with anti- CD103 (diluted in TNB buffer, 10 μg/ml) for 1 h at room temperature or overnight at 4 °C. The sections were washed thoroughly in washing buffer three times and goat anti-rat IgG (10 μg/ml, Invitrogen) was added for 1 h at room temperature. After washing the sections were incubated with fluorescent FITC Tyramide Amplification reagent for 5 min in the dark at room temperature. After washing three times, the sections were carefully dried and the CD11c-biotin antibody (1 μg/ml), (eBioscience, Hatfield, UK) added to sections and incubated for 1 h at room temperature. After washing, streptavidin–horseradish peroxidase (HRP, 1.5 μg/ml), (Invitrogen, Cheshire, UK) was applied and the sections incubated for 30 min at room temperature in the dark. After washing thoroughly, the sections were incubated with cyanine3 Tyramide Amplification System (Perkin Elmer) for 5 min at room temperature in the dark. After a final wash, the tissue sections were mounted using a mounting media containing DAPI (Vectashield, Vector Laboratories, Peterborough, UK) and then sealed until viewing. Slides were analysed under an Olympus BX51 fluorescence microscope equipped with lenses at × 20 magnification (Olympus, Japan). Cells were counted per field of view in a blind and randomised order. Three fields/views were counted per section. Control sections were prepared with only secondary antibodies but no primary antibody.

### Statistics and mathematical analysis

Statistical analysis was performed using Mann- Whitney U test or Kruskal-Wallis as appropriate. The *P*-values of < 0.05 were considered significant. All statistical analyses were carried out using Graph Pad Prism for mac, version 6 and 7 (La Jolla, CA). Data are expressed as mean ± SEM.

The data that support the findings of this study are available from the corresponding author upon reasonable request.

## Supplementary Information

**Additional file 1.**

**Additional file 2.**

## References

[1] Varol C, Vallon-Eberhard A, Elinav E, Aychek T, Shapira Y, Luche H (2009). Intestinal lamina propria dendritic cell subsets have different origin and functions. Immunity..

[2] Vicente-Manzanares M, Choi CK, Horwitz AR (2009). Integrins in cell migration--the actin connection. J Cell Sci.

[3] Evans R, Patzak I, Svensson L, De Filippo K, Jones K, McDowall A (2009). Integrins in immunity. J Cell Sci.

[4] Huttenlocher A, Horwitz AR (2011). Integrins in cell migration. Cold Spring Harb Perspect Biol.

[5] Abram CL, Lowell CA (2009). The ins and outs of leukocyte integrin signaling. Annu Rev Immunol.

[6] Solovjov DA, Pluskota E, Plow EF (2005). Distinct roles for the alpha and beta subunits in the functions of integrin alphaMbeta2. J Biol Chem.

[7] Zarbock A (2013). beta2-integrin activity: the role of thiols. Blood..

[8] Morrison VL, James MJ, Grzes K, Cook P, Glass DG, Savinko T (2014). Loss of beta2-integrin-mediated cytoskeletal linkage reprogrammes dendritic cells to a mature migratory phenotype. Nat Commun.

[9] von Andrian UH, Chambers JD, McEvoy LM, Bargatze RF, Arfors KE, Butcher EC (1991). Two-step model of leukocyte-endothelial cell interaction in inflammation: distinct roles for LECAM-1 and the leukocyte beta 2 integrins in vivo. Proc Natl Acad Sci U S A.

[10] Li Z (1999). The alphaMbeta2 integrin and its role in neutrophil function. Cell Res.

[11] Yakubenko VP, Belevych N, Mishchuk D, Schurin A, Lam SC, Ugarova TP (2008). The role of integrin alpha D beta2 (CD11d/CD18) in monocyte/macrophage migration. Exp Cell Res.

[12] Hogg N, Stewart MP, Scarth SL, Newton R, Shaw JM, Law SK (1999). A novel leukocyte adhesion deficiency caused by expressed but nonfunctional beta2 integrins mac-1 and LFA-1. J Clin Invest.

[13] Marski M, Kandula S, Turner JR, Abraham C (2005). CD18 is required for optimal development and function of CD4(+) CD25(+) T regulatory cells. J Immunol.

[14] Lammermann T, Bader BL, Monkley SJ, Worbs T, Wedlich-Soldner R, Hirsch K (2008). Rapid leukocyte migration by integrin-independent flowing and squeezing. Nature..

[15] Jang MH, Sougawa N, Tanaka T, Hirata T, Hiroi T, Tohya K (2006). CCR7 is critically important for migration of dendritic cells in intestinal lamina propria to mesenteric lymph nodes. J Immunol.

[16] Cruickshank SM, Deschoolmeester ML, Svensson M, Howell G, Bazakou A, Logunova L (2009). Rapid dendritic cell mobilization to the large intestinal epithelium is associated with resistance to Trichuris muris infection. J Immunol (Baltimore, Md : 1950).

[17] Bowcutt R, Bramhall M, Logunova L, Wilson J, Booth C, Carding SR (2014). A role for the pattern recognition receptor Nod2 in promoting recruitment of CD103+ dendritic cells to the colon in response to Trichuris muris infection. Mucosal Immunol.

[18] Huang SW, Walker C, Pennock J, Else K, Muller W, Daniels MJ (2017). P2X7 receptor-dependent tuning of gut epithelial responses to infection. Immunol Cell Biol.

[19] Gawden-Bone C, West MA, Morrison VL, Edgar AJ, McMillan SJ, Dill BD (2014). A crucial role for beta2 integrins in podosome formation, dynamics and toll-like-receptor-signaled disassembly in dendritic cells. J Cell Sci.

[20] Bancroft AJ, Else KJ, Grencis RK (1994). Low-level infection with Trichuris-Muris significantly affects the polarization of the Cd4 response. Eur J Immunol.

[21] Bancroft AJ, Else KJ, Humphreys NE, Grencis RK (2001). The effect of challenge and trickle Trichuris muris infections on the polarisation of the immune response. Int J Parasitol.

[22] Bowcutt R, Forman R, Glymenaki M, Carding SR, Else KJ, Cruickshank SM (2014). Heterogeneity across the murine small and large intestine. World J Gastroenterol.

[23] Kim YS, Ho SB (2010). Intestinal goblet cells and mucins in health and disease: recent insights and progress. Curr Gastroenterol Rep.

[24] Hasnain SZ, McGuckin MA, Grencis RK, Thornton DJ (2012). Serine protease(s) secreted by the nematode Trichuris muris degrade the mucus barrier. PLoS Negl Trop Dis.

[25] Hasnain SZ, Thornton DJ, Grencis RK (2011). Changes in the mucosal barrier during acute and chronic Trichuris muris infection. Parasite Immunol.

[26] Ohl L, Mohaupt M, Czeloth N, Hintzen G, Kiafard Z, Zwirner J (2004). CCR7 governs skin dendritic cell migration under inflammatory and steady-state conditions. Immunity..

[27] Diao J, Winter E, Chen W, Cantin C, Cattral MS (2004). Characterization of distinct conventional and plasmacytoid dendritic cell-committed precursors in murine bone marrow. J Immunol.

[28] Bogunovic M, Ginhoux F, Helft J, Shang LM, Hashimoto D, Greter M (2009). Origin of the Lamina Propria dendritic cell network. Immunity..

[29] Persson EK, Scott CL, Mowat AM, Agace WW (2013). Dendritic cell subsets in the intestinal lamina propria: ontogeny and function. Eur J Immunol.

[30] Varol C, Landsman L, Fogg DK, Greenshtein L, Gildor B, Margalit R (2007). Monocytes give rise to mucosal, but not splenic, conventional dendritic cells. J Exp Med.

[31] Bain CC, Schridde A (2018). Origin, differentiation, and function of intestinal macrophages. Front Immunol.

[32] Souza COS, Espindola MS, Fontanari C, Prado MKB, Frantz FG, Rodrigues V (2018). CD18 regulates monocyte hematopoiesis and promotes resistance to experimental Schistosomiasis. Front Immunol.

[33] Semmrich M, Plantinga M, Svensson-Frej M, Uronen-Hansson H, Gustafsson T, Mowat AM (2012). Directed antigen targeting in vivo identifies a role for CD103+ dendritic cells in both tolerogenic and immunogenic T-cell responses. Mucosal Immunol.

[34] Everts B, Tussiwand R, Dreesen L, Fairfax KC, Huang SC, Smith AM (2016). Migratory CD103+ dendritic cells suppress helminth-driven type 2 immunity through constitutive expression of IL-12. J Exp Med.

[35] Cerovic V, Houston SA, Scott CL, Aumeunier A, Yrlid U, Mowat AM (2013). Intestinal CD103(−) dendritic cells migrate in lymph and prime effector T cells. Mucosal Immunol.

[36] Fujimoto K, Karuppuchamy T, Takemura N, Shimohigoshi M, Machida T, Haseda Y (2011). A new subset of CD103(+)CD8 alpha(+) dendritic cells in the small intestine expresses TLR3, TLR7, and TLR9 and induces Th1 response and CTL activity. J Immunol.

[37] Tang H, Cao WP, Kasturi SP, Ravindran R, Nakaya HI, Kundu K (2010). The T helper type 2 response to cysteine proteases requires dendritic cell-basophil cooperation via ROS-mediated signaling. Nat Immunol.

[38] Oliphant CJ, Hwang YY, Walker JA, Salimi M, Wong SH, Brewer JM (2014). MHCII-mediated dialog between group 2 innate lymphoid cells and CD4(+) T cells potentiates type 2 immunity and promotes parasitic Helminth expulsion. Immunity..

[39] Nakanishi K (2010). Basophils as APC in Th2 response in allergic inflammation and parasite infection. Curr Opin Immunol.

[40] Wynn TA (2009). Basophils trump dendritic cells as APCs for T(H)2 responses. Nat Immunol.

[41] Klementowicz JE, Travis MA, Grencis RK (2012). Trichuris muris: a model of gastrointestinal parasite infection. Semin Immunopathol.

[42] Travis MA, Reizis B, Melton AC, Masteller E, Tang Q, Proctor JM (2007). Loss of integrin alpha(v)beta8 on dendritic cells causes autoimmunity and colitis in mice. Nature..

[43] Savinko TS, Morrison VL, Uotila LM, Wolff CHJ, Alenius HT, Fagerholm SC (2015). Functional Beta2-Integrins restrict skin inflammation in vivo. J Invest Dermatol.

[44] Arnaout MA (2016). Biology and structure of leukocyte beta 2 integrins and their role in inflammation. F1000Res.

[45] Grabbe S, Varga G, Beissert S, Steinert M, Pendl G, Seeliger S (2002). Beta2 integrins are required for skin homing of primed T cells but not for priming naive T cells. J Clin Invest.

[46] Morrison VL, MacPherson M, Savinko T, Lek HS, Prescott A, Fagerholm SC (2013). The beta2 integrin-kindlin-3 interaction is essential for T-cell homing but dispensable for T-cell activation in vivo. Blood..

[47] Varga G, Balkow S, Wild MK, Stadtbaeumer A, Krummen M, Rothoeft T (2007). Active MAC-1 (CD11b/CD18) on DCs inhibits full T-cell activation. Blood..

[48] Haasken S, Auger JL, Binstadt BA (2011). Absence of beta (2) Integrins impairs regulatory T cells and exacerbates CD4(+) T cell-dependent autoimmune Carditis. J Immunol.

[49] Ehirchiou D, Xiong Y, Xu GW, Chen WJ, Shi YF, Zhang L (2007). CD11b facilitates the development of peripheral tolerance by suppressing Th17 differentiation. J Exp Med.

[50] Moutsopoulos NM, Konkel J, Sarmadi M, Eskan MA, Wild T, Dutzan N (2014). Defective neutrophil recruitment in leukocyte adhesion deficiency type I disease causes local IL-17-driven inflammatory bone loss. Sci Transl Med.

[51] Cliffe LJ, Grencis RK (2004). The Trichuris muris system: a paradigm of resistance and susceptibility to intestinal nematode infection. Adv Parasitol.

[52] Cliffe LJ, Humphreys NE, Lane TE, Potten CS, Booth C, Grencis RK (2005). Accelerated intestinal epithelial cell turnover: a new mechanism of parasite expulsion. Science..

[53] Faulkner H, Renauld JC, Van Snick J, Grencis RK (1998). Interleukin-9 enhances resistance to the intestinal nematode Trichuris muris. Infect Immun.

[54] Watt FM (2002). Role of integrins in regulating epidermal adhesion, growth and differentiation. EMBO J.

[55] McDole JR, Wheeler LW, McDonald KG, Wang B, Konjufca V, Knoop KA (2012). Goblet cells deliver luminal antigen to CD103+ dendritic cells in the small intestine. Nature..

[56] Else KJ, Wakelin D, Wassom DL, Hauda KM (1990). The influence of genes mapping within the major histocompatibility complex on resistance to Trichuris muris infections in mice. Parasitology..

[57] Bowcutt R, Bell LV, Little M, Wilson J, Booth C, Murray PJ (2011). Arginase-1-expressing macrophages are dispensable for resistance to infection with the gastrointestinal helminth Trichuris muris. Parasite Immunol.

[58] Else KJ, Grencis RK (1991). Cellular immune responses to the murine nematode parasite Trichuris muris. I. Differential cytokine production during acute or chronic infection. Immunology..

[59] Hansson GC, Johansson ME. The inner of the two Muc2 mucin-dependent mucus layers in colon is devoid of bacteria. Gut Microbes. 2010;1(1):51–4. 10.4161/gmic.1.1.10470 PMC3035142 21327117

