# Supplementary figures and images for: The role of β2 integrin in dendritic cell migration during infection
Source: BMC Immunol. 2021 Jan 6;22:2. doi: 10.1186/s12865-020-00394-5 (PMC7789014; doi:10.1186/s12865-020-00394-5)

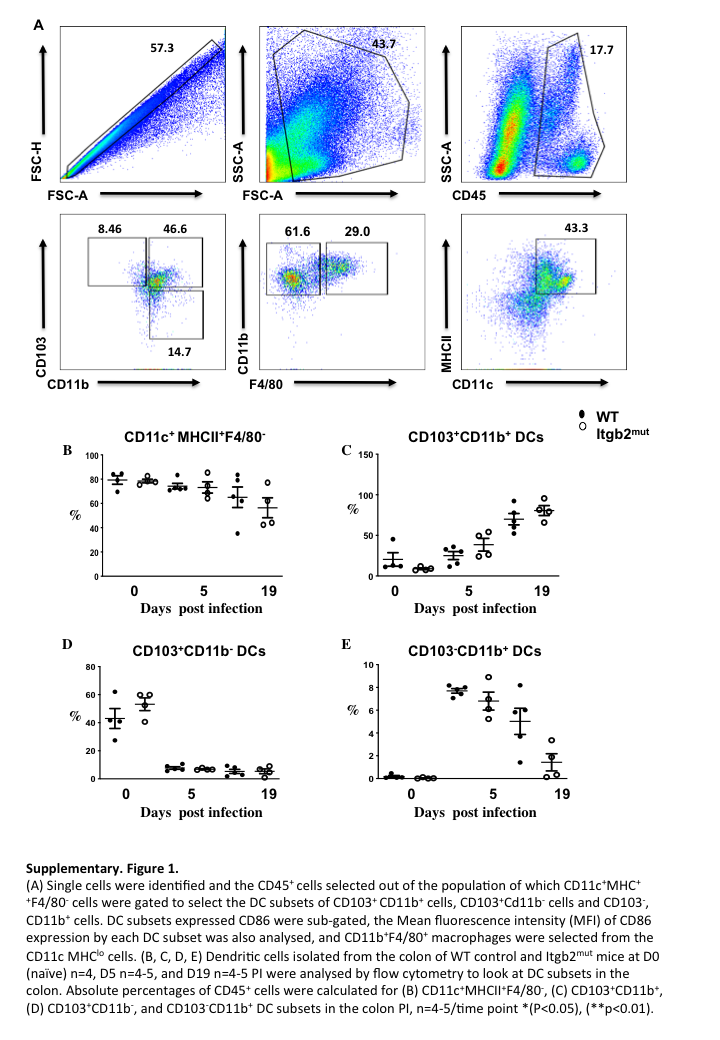

Supplement: Supplementary file 1 — Additional file 1. [file 12865_2020_394_MOESM1_ESM.tiff]

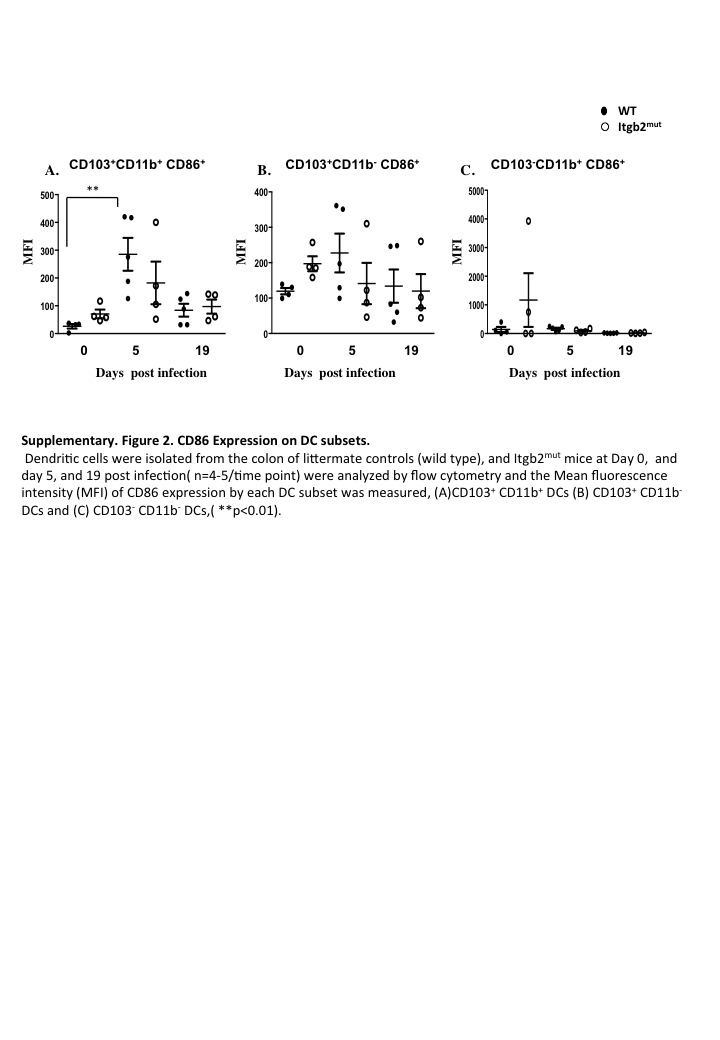

Supplement: Supplementary file 2 — Additional file 2. [file 12865_2020_394_MOESM2_ESM.tiff]
